# Supplementary material for: Real-time prediction of COVID-19 related mortality using electronic health records
Source: Nat Commun. 2021 Feb 16;12:1058. doi: 10.1038/s41467-020-20816-7 (PMC7886884; doi:10.1038/s41467-020-20816-7)
Supplement: Supplementary file 1 — Supplementary Information [file 41467_2020_20816_MOESM1_ESM.pdf]

Supplementary Information for

Real-time Prediction of COVID-19 related Mortality using Electronic Health Records

Patrick Schwab, Arash Mehrjou, Sonali Parbhoo, Leo Anthony Celi, Jürgen Hetzel, Markus Hofer, Bernhard Schölkopf, Stefan Bauer

S Supplementary Information

1 Subcohort Analysis with Fewer Missing Covariates

Since our analysis was based on data collected in real-world care settings, certain covariates, including Fibrin D-dimer, hsCRP, Gamma Glutamyl Transferase (GGT) and IL-6, were missing in a large fraction of patients (Table 1). In the evaluation presented in the main body of this work, we imputed all missing covariates using MICE prior to calculating risk predictions (Section "Preprocessing"). However, there may have been differences between the true unmeasured underlying value of those covariates and the value estimated by MICE, and any potential imputation errors propagate to the prediction system that predicts mortality risk based on those imputed covariates. While all prediction systems received input data that was imputed using the same methodology, imputation errors may potentially have disproportionally affected risk prediction systems that rely on a small number of rarely sampled covariates. For example, Yan et al. [17] relies strongly on hsCRP which is rarely available in patients' EHRs (Table 1). To ensure risk prediction systems were fairly evaluated, we performed further analyses on six additional subcohorts of the Optum test set consisting of patients (i) for which the four covariates with the highest missingness ratios (Fibrin D-dimer, hsCRP, GGT, IL-6) were available at least once in their EHR (Supplementary Table 7), and (ii) for which a maximum of respectively 6 and 9 covariates[1] were not available in their EHR (Supplementary Table 8). We note that the six additional subcohorts with fewer missing covariates were affected markedly by sampling bias. In particular, mortality rates were significantly higher in the subcohorts with fewer missing covariates compared to the overall cohort - potentially stemming from severe patients having longer durations of stay which, in turn, increased the time during which tests could be performed on those patients, and from more complex cases being tested more thoroughly to conclusively establish their underlying condition. While extrapolation beyond these subcohorts is difficult due to their non-representative nature, the results of the analysis showed that the use of missingness imputation did not disproportionally disadvantage any included prediction system since the overall ranking of prediction systems and their relative predictive performance differences were, with few exceptions, sustained in the subcohorts with fewer missing covariates - where missingness imputation was either used more sparingly or not at all for certain covariates (Supplementary Figure 6).

2 Evaluating Risk Predictors under Missingness

There are four main approaches to comparing risk predictors that rely on different sets of covariates under missingness.

[1] We chose 6 missing covariates as the lower cohort cutoff to maintain a meaningful sample size, and 9 as the higher cutoff to have a comparison cohort with a slightly higher missingness in order to surface any potential patterns related to increased missingness.

Option 1: Evaluate on all patients, impute missing information (our chosen approach). In this evaluation approach, all risk predictors are evaluated on all COVID-19 patients, irrespective of whether any of their covariates may have originally been missing. Missing covariates are imputed using MICE - the recommended standard approach to handling missingness in this setting [28] - and the predictors therefore receive a full set of covariates for all patients. We note that there may be differences between the true underlying value of a covariate and the covariate value imputed using MICE. However, because all predictors receive input covariates that were imputed in the same manner, they are all impacted in the same way by potential imputation errors - making Option 1 fair to all predictors, since no system can gain an undue advantage over any other since neither has access to the true underlying values.

Option 2: Evaluate on all patients, do not predict when required covariates not available. Option 2 corresponds to a clinical use pattern in which a risk predictor is only used when all required covariates are available (if the system is not designed to handle missing information). Using Option 2, the predictive performance in terms of sensitivity of predictors not designed to handle missingness would be strictly lower than the one calculated using Option 1 since they would not be able to detect mortality events for patients for which required covariates are missing. Option 2 is therefore not as representative as Option 1 of the performance of a predictor that could be expected in a real-world care setting because it does not give all system equal opportunity to identify mortality events in the same patients.

Option 3: Evaluate only on patients with all data available. Evaluating only on complete cases may misstate the predictive performance of risk predictors by evaluating them only on a non-representative subset of patients. Further, the relevant subset of patients (each with potentially their own unique sampling bias) is different for each prediction system since they have different sets of required covariates. Option 3 makes comparisons of multiple predictors in a representative manner difficult, since the union of required covariates for all compared systems reduces the number of leftover patients down to a very small, non-representative cohort that would likely be strongly affected by sampling bias. In addition, Option 3 is also problematic because predicting mortality for the subset of patients that have all required covariates available may be easier or harder than for the overall population of COVID-19 patients - therefore giving a misleading picture of the predictive performance that could be expected in a real-world care setting.

Option 4: Do not compare performance at all on datasets with missingness. Option 4 would imply that the performance of prediction systems can not be compared in real-world settings, since missingness is characteristic for real-world care settings (Supplementary Table 1). Given that the real-world performance of risk predictors is of high scientific and practical interest, we

believe that Option 4 is not a valid option.

Among the main evaluation options, we therefore chose Option 1 as the most representative evaluation approach for the envisioned clinical use of predictors of COVID-19 related mortality.

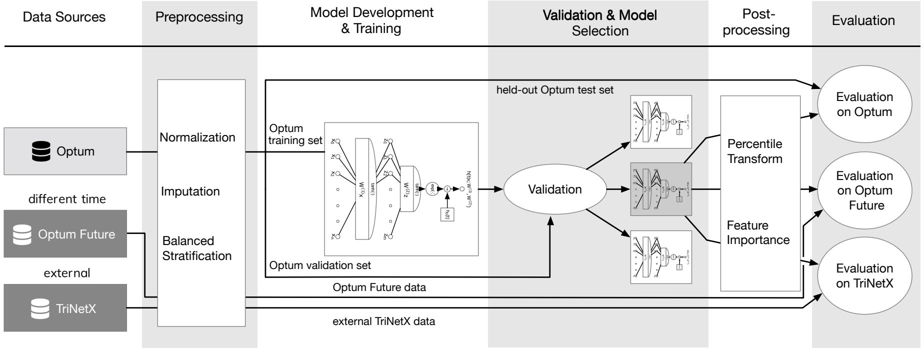

Supplementary Figure 1: During training the model is provided with EHR data, including lab tests, clinical measurements, and information about pre-existing conditions. Before the data is fed to the model, the preprocessing phase handles missing values and standardises scaling of each covariate (Section "Preprocessing"). CovEWS was trained on the training fold of the Optum cohort, and evaluated on the held-out Optum test cohort, the Optum Future cohort and the external TriNetX cohort (Section "Data Collection"). The proposed model accommodates the effect of time-varying and nonlinearly interacting covariates and is trained using partial likelihood as detailed in Section "Algorithm Details". Section "Preprocessing" presents further details on preprocessing.

Supplementary Table 1: Comparison of the percentage of COVID-19 diagnosed patients without (w/o) a recorded SARS-CoV-2 test result across the analysed datasets (Optum, Optum Future, TriNetX). The higher fraction of COVID-19 diagnoses without a recorded SARS-CoV-2 test result in the Optum cohort compared to the Optum Future cohort is likely a result of the available testing resources having been more scarce early on in the pandemic. Since the Optum database indicates a generally high testing ratio substantiating COVID-19 diagnoses, the relatively higher percentage of COVID-19 diagnoses without corresponding tests in TriNetX is likely a result of inconsistent coding of SARS-CoV-2 tests early in the pandemic.

	Optum	Optum Future	TriNetX
COVID-19 diagnoses w/o test results [%]	6.31	0.00	37.92

Supplementary Table 2: Descriptions, Logical Observation Identifiers Names and Codes (LOINC) codes (if available), and p-values for each input covariate used by CovEWS. The p-values were calculated using Wald's χ^2 tests for effect (H_0 = no effect) of the coefficients in CovEWS (linear) corresponding to the respective input covariates on the Optum training cohort.

Covariate	Description	p-value
Sex	-	0.41
Age	-	< 0.005
Weight	-	0.92
Height	-	0.80
Body Mass Index (BMI)	-	0.85
Intubation	Whether or not the patient is intubated	0.04
Temperature	Body temperature (LOINC: 8310-5 on TriNetX)	0.89
SpO_2	Oxygen saturation by pulse oximetry (LOINC: 59408-5)	< 0.005
Heart rate	-	0.75
Respiratory rate	-	0.32
Systolic blood pressure	-	0.01
Diastolic blood pressure	-	< 0.005
Kidney disease	see Supplementary Table 3	0.05
Ischemic heart disease	see Supplementary Table 3	0.04
Other heart disease	see Supplementary Table 3	0.01
Cerebrovascular disease	see Supplementary Table 3	0.20
Hypertension	see Supplementary Table 3	0.03
Diabetes	see Supplementary Table 3	0.15
Hyperlipidemia	see Supplementary Table 3	0.17
Cancer	see Supplementary Table 3	0.65
Dyspnea	see Supplementary Table 3	0.42
COPD	see Supplementary Table 3	0.18
Asthma	see Supplementary Table 3	0.80
Pulmonary embolism	see Supplementary Table 3	0.58
Connective tissue disease	see Supplementary Table 3	0.84
Inflammatory bowel disease	see Supplementary Table 3	0.99
Osteoarthritis	see Supplementary Table 3	0.37
Rheumatoid arthritis	see Supplementary Table 3	0.79
HIV	see Supplementary Table 3	0.97
Smoking (never)	-	0.81
Smoking (previous)	-	0.61
Smoking (current)	-	0.84
Smoking (unknown)	-	0.85
White blood cells	White blood cell count (LOINC: 26464-8)	< 0.005

Supplementary Table 2 cont.

Neutrophil	Neutrophils per 100 leukocytes in blood (LOINC: 26511-6)	< 0.005
Lymphocytes	Lymphocytes per 100 leukocytes in blood (LOINC: 26478-8)	< 0.005
Eosinophil	Eosinophils per 100 leukocytes in blood (LOINC: 26450-7)	0.38
Basophil	Basophils per 100 leukocytes in blood (LOINC: 30180-4)	0.33
Platelets	Platelets [#/volume] in blood (LOINC: 26515-7)	0.28
C-reactive protein	C-reactive protein [mass/volume] in serum or plasma (LOINC: 1988-5)	0.52
hs. C-reactive protein	C-reactive protein [mass/volume] in serum or plasma by high sensitivity method (LOINC: 30522-7)	< 0.005
Procalcitonin	Procalcitonin [mass/volume] in serum or plasma (LOINC: 33959-8)	0.46
Fibrin D-dimer	Fibrin D-dimer Fibrinogen Equivalent Units (FEU) [mass/volume] in platelet poor plasma	0.13
Ferritin	Ferritin [mass/volume] in serum or plasma (LOINC: 2276-4)	0.12
Cardiac troponin T	Cardiac troponin T [mass/volume] in serum or plasma (LOINC: 6598-7)	0.42
Creatinine	Creatinine [mass/volume] in Serum or Plasma (LOINC: 2160-0)	0.03
Lactate dehydrogenase	Lactate dehydrogenase [enzymatic activity/volume] in serum or plasma (LOINC: 2532-0, 14804-9)	0.01
Gamma glutamyl transferase	Gamma glutamyl transferase [enzymatic activity/volume] in serum or plasma (LOINC: 2324-2)	0.69
Aspartate aminotransferase	Aspartate aminotransferase [enzymatic activity/volume] in serum or plasma (LOINC: 1920-8)	0.10
Creatine kinase	Creatine kinase enzymatic activity/volume] in serum or plasma (LOINC: 2157-6)	0.60

Supplementary Table 2 cont.

Bilirubin	Bilirubin [mass/volume] in serum or plasma (LOINC: 1975-2)	0.28
Albumin	Albumin [mass/volume] in serum or plasma (LOINC: 1751-7)	< 0.005
Interleukin 6 (IL-6)	Interleukin 6 [mass/volume] in serum or plasma (LOINC: 26881-3)	0.68
pH	pH of blood (LOINC: 2744-1, 2746-6)	< 0.005
PCO_2	Carbon dioxide [partial pressure] in arterial blood (LOINC: 2019-8)	0.02
PaO_2	Oxygen [partial pressure] in arterial blood (LOINC: 2703-7)	0.63
HCO_3	Bicarbonate [moles/volume] in blood (LOINC: 1959-6, 1960-4)	0.17
CO_2	Carbon dioxide, total [moles/volume] in serum or plasma (LOINC: 2028-9)	0.21

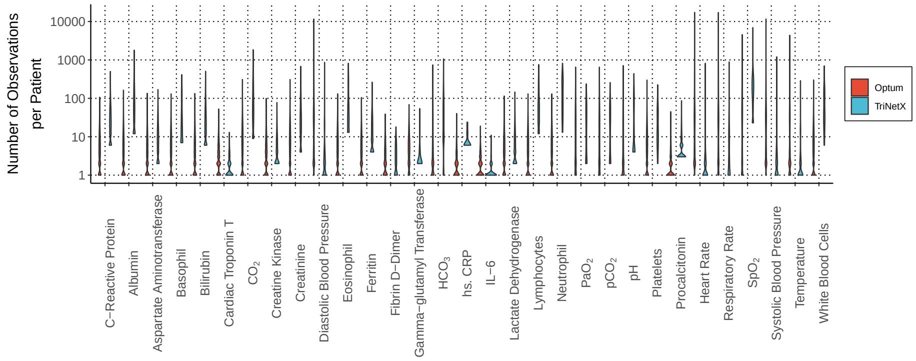

Supplementary Figure 2: Distributions of the number of observations per patient (y-axis, log scale, violin plots) for time-varying covariates (x-axis) in the Optum and TriNetX datasets for patients that have at least one observation of a given covariate. The percentage of patients with no observation for each covariate is shown in Supplementary Table 1.

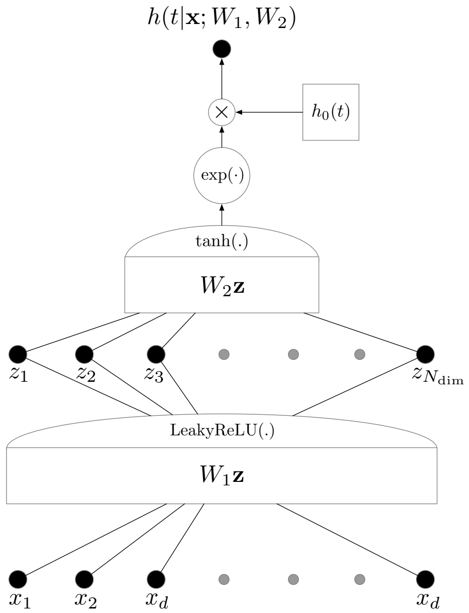

Supplementary Figure 3: Schematic illustration of a neural network realisation of the nonlinear exponent of the time-varying Cox hazard function with $L = 1$ hidden layers with N_{dim} hidden units and one output layer.

Supplementary Table 3: International Statistical Classification of Diseases and Related Health Problems (ICD) codes corresponding to the disease classifications and symptoms used in our analysis. HIV = Human Immunodeficiency Virus, COPD = Chronic Obstructive Pulmonary Disease.

Disease classification	ICD-9	ICD-10
Kidney disease	585 - 586	N18 - N19
Ischemic heart disease	410 - 414	I20 - I25
Other heart disease	390 - 398, 401 - 405, 416 - 417, 420 - 429, 115.03, 115.04, 115.13, 115.14, 115.93, 115.94	I27 - I52
Cerebrovascular disease	430 - 434	I60 - I69
Hypertension	401 - 405	I10 - I15
Diabetes	249 - 250, 357.2, 366.41	E10 - E14
Hyperlipidemia	272	E78
Cancer	140 - 239	C
Dyspnea	786.1, 786.2, 786.8, 786.9	R06
COPD	496, 491.21, 491.22	J44
Asthma	493	J45
Pulmonary embolism	415	I26
Connective tissue disease	446, 710 - 711, 713, 725, 136.1, 279.8, 517.2, 728.5	I30 - I36
Inflammatory bowel disease	555 - 556	K50 - K51
Osteoarthritis	715	M15 - M19
Rheumatoid arthritis	274, 712 - 714, 716, 719	M05 - M14
HIV	42	B20 - B24

Supplementary Table 4: Comparison of missingness (in %), i.e. the fraction of patients that did not have any record for a specific covariate in their EHR, in several important clinical covariates between the Optum test set and the non-hospitalised subgroup of the Optum test set. SBP = Systolic Blood Pressure, RR = Respiratory rate, WBC = White blood cells, CRP = C-reactive protein.

Cohort	SBP	RR	WBC	CRP	Albumin	Platelets
Optum Test Set	38.56	42.21	48.40	67.81	53.48	48.45
Non-hospitalised	54.00	59.57	64.67	81.10	69.02	64.72

Supplementary Table 5: Hyperparameter ranges used for hyperparameter optimisation of CovEWS and CovEWS (linear). Comma-delimited lists indicate discrete choices with equal selection probability. Hyperparameters selected after hyperparameter optimisation (Section “Hyperparameter Optimisation”) are highlighted in bold.

	Hyperparameter	Range / Choices
Linear	Regularisation strength λ	0.01, **0.1**, 1.0
CovEWS	Number of layers L	**1**, 2
	Number of hidden units N_{dim}	64, **128**, 256
	Dropout percentage p_{dropout}	10%, **20%**

Supplementary Table 6: Optimal thresholds of CovEWS scores to maximise specificity at greater than 85%, 90% and 95% sensitivity (Sens.) for each prediction horizon as selected on their respective receiver operator characteristic (ROC) curves on the held-out Optum test set.

Sens.	1 hour	2 hours	4 hours	8 hours	16 hours	24 hours	48 hours	96 hours	192 hours
85%	61	62	56	54	51	48	45	39	36
90%	44	44	42	41	39	38	38	32	27
95%	34	34	33	31	28	27	27	22	19

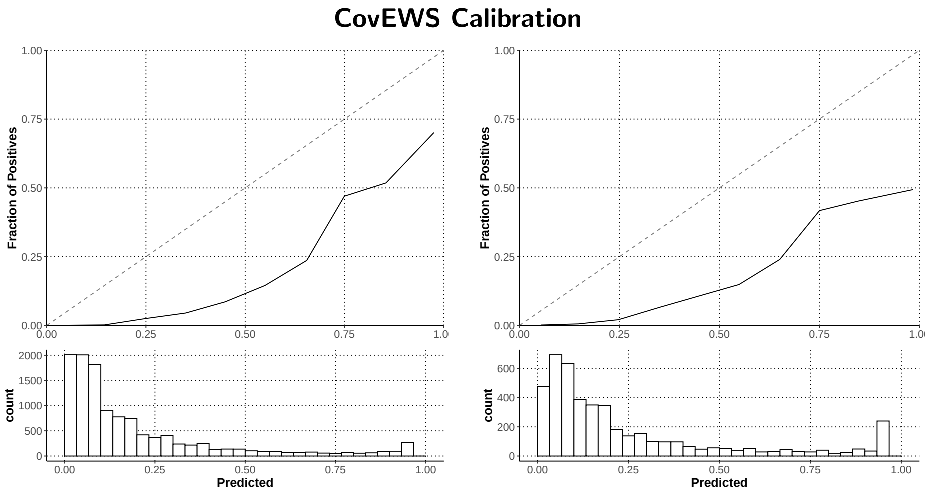

(a) Held-out Optum Test Set

(b) External TriNetX Test Set

Supplementary Figure 4: Calibration plots and counts of observed predictions of CovEWS on the held-out Optum test set (left) and the external TriNetX test set (right) when interpreting the predicted risk percentile of CovEWS as the probability of a mortality event being observed within the next 24 hours. The reference time point for those patients that did not have an observed mortality event is the date of their respective last observed EHR entry. When interpreted as a patient's mortality probability, CovEWS overestimates the risk since it can not account a priori for clinical interventions and potential future patient trajectory changes that may occur rapidly and frequently. Direct interpretation of CovEWS scores as a mortality probability is discouraged, and CovEWS scores should instead be seen as a relative risk score to stratify patients into risk groups.

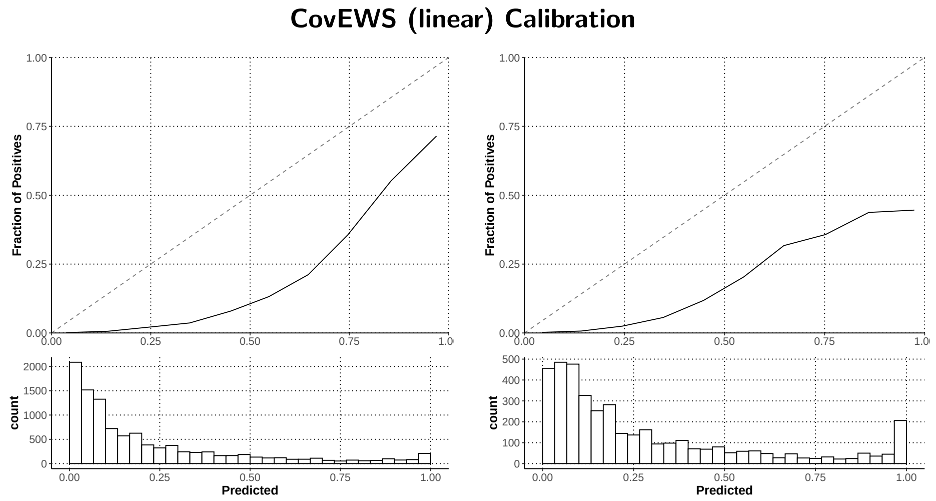

(a) Held-out Optum Test Set

(b) External TriNetX Test Set

Supplementary Figure 5: Calibration plots and counts of observed predictions of CovEWS (linear) on the held-out Optum test set (left) and the external TriNetX test set (right) when interpreting the predicted risk percentile of CovEWS (linear) as the probability of a mortality event being observed within the next 24 hours. The reference time point for those patients that did not have an observed mortality event is the date of their respective last observed EHR entry. Like the version of CovEWS that models non-linear interactions, CovEWS (linear) overestimates mortality risk when interpreted as a patient's mortality probability.

Supplementary Table 7: Descriptive statistics for selected subgroups consisting of held-out Optum test cohort patients with certain covariates (Fibrin D-dimer, hsCRP, GGT, IL-6) available at least once in their EHRs. Input covariates of CovEWS are placed towards the bottom of the table and separated from covariates that are not inputs by a horizontal line. For binary covariates, the Value columns indicate the percentage of patients presenting with the condition at the end of their respective observation periods. For continuous measurements, the Value columns indicate the median and 10th and 90th percentiles in parentheses of the observed value for measurements that are collected once per patient, such as age, and the median of observed values for measurements that are collected multiple times per patient, such as heart rate. BMI = Body Mass Index, HIV = Human Immunodeficiency Virus, COPD = Chronic Obstructive Pulmonary Disease, GGT = Gamma Glutamyl Transferase, AAT = Aspartate Aminotransferase, IL6 = Interleukin 6, n/a = not available

	Optum Test Set March 21 - June 5 2020							
	Fibrin D-dimer		hsCRP		GGT		IL-6	
	Value	Miss.%	Value	Miss.%	Value	Miss.%	Value	Miss.%
Patients [#]	972	-	525	-	471	-	640	-
COVID-19 [%]	100.00	-	100.00	-	100.00	-	100.00	-
Hispanic [%]	18.11	-	15.24	-	7.43	-	7.66	-
Black [%]	23.87	-	28.76	-	10.83	-	14.53	-
Caucasian [%]	54.12	-	45.90	-	38.85	-	44.84	-
Asian [%]	2.57	-	3.05	-	7.22	-	9.38	-
Hospital admission [%]	77.98	-	59.24	-	68.37	-	50.47	-
ICU admission [%]	23.46	-	16.00	-	1.91	-	0.31	-
Mortality [%]	12.65	-	13.33	-	17.20	-	17.97	-
**Model Inputs**								
Female [%]	47.84	-	49.14	-	40.55	-	35.16	-
Age [years]	62.00 (37.00, 83.00)	-	63.00 (36.00, 84.00)	-	62.00 (34.00, 83.00)	-	65.00 (42.00, 84.00)	-
Weight [kg]	83.92 (60.01, 119.27)	13.07	86.18 (60.29, 119.79)	10.67	76.47 (58.95, 105.20)	12.74	79.50 (59.35, 110.38)	7.03
Height [cm]	167.63 (152.40, 182.88)	15.43	167.64 (154.92, 182.88)	15.05	167.64 (154.90, 180.34)	19.75	167.64 (154.94, 182.88)	13.44
BMI [kg/m^2]	29.83 (22.33, 42.80)	15.84	30.32 (23.00, 42.02)	11.62	27.08 (21.38, 36.96)	15.50	27.98 (22.04, 38.10)	8.59
Intubation [%]	11.42	-	12.57	-	16.56	-	25.31	-
Temperature [C]	36.95 (36.55, 37.53)	8.95	36.83 (36.46, 37.41)	11.43	36.90 (36.50, 37.61)	6.79	36.80 (36.40, 37.59)	8.91
SpO_2 [%]	95.39 (93.02, 97.90)	3.60	95.29 (92.97, 97.67)	3.62	95.12 (92.01, 98.25)	4.46	94.26 (91.86, 96.48)	5.78
Heart rate [/min]	85.03 (69.90, 100.83)	10.60	83.88 (70.68, 100.16)	8.95	88.14 (71.52, 104.47)	3.18	87.40 (73.18, 103.62)	3.28
Respiratory rate [/min]	19.66 (17.00, 25.84)	9.77	19.55 (17.46, 24.38)	9.71	20.37 (17.49, 26.41)	5.52	21.10 (18.54, 26.51)	5.62
Dyspnea [%]	66.15	-	79.43	-	69.43	-	80.94	-
Sys. blood pressure [mmHg]	124.00 (108.08, 145.82)	9.77	123.84 (109.17, 143.74)	8.76	120.75 (104.62, 139.68)	3.18	121.21 (106.74, 138.82)	3.28
Dias. blood pressure [mmHg]	70.48 (60.55, 82.35)	9.88	70.89 (60.87, 82.90)	8.76	69.84 (61.09, 82.44)	3.18	70.42 (61.27, 80.75)	3.28
Kidney disease [%]	22.53	-	27.24	-	23.14	-	27.50	-
Ischemic heart disease [%]	26.03	-	33.71	-	37.15	-	38.91	-
Other heart diseases [%]	77.67	-	80.76	-	74.73	-	81.56	-
Cerebovascular disease [%]	17.70	-	17.33	-	18.05	-	14.84	-
Hypertension [%]	69.24	-	71.24	-	60.51	-	65.62	-
Diabetes [%]	40.95	-	45.52	-	38.43	-	42.03	-
Hyperlipidemia [%]	58.95	-	57.52	-	48.41	-	52.50	-
Cancer [%]	31.07	-	24.38	-	13.16	-	14.69	-
COPD [%]	17.39	-	15.05	-	9.34	-	8.59	-
Asthma [%]	18.31	-	19.81	-	12.74	-	12.19	-
Pulmonary embolism [%]	4.84	-	7.05	-	6.16	-	6.09	-
Connective tissue disease [%]	4.94	-	4.57	-	1.49	-	2.97	-
Inflamatory bowel disease [%]	1.75	-	1.52	-	1.27	-	2.03	-
Osteoarthritis [%]	31.58	-	32.38	-	19.75	-	21.41	-
Rheumatroid arthritis [%]	38.58	-	36.00	-	12.10	-	15.00	-
HIV [%]	0.21	-	1.71	-	1.49	-	0.47	-
Smoking (never)	54.01	16.36	57.14	16.57	63.69	14.86	64.22	15.16
Smoking (previous)	22.84	16.36	21.52	16.57	17.41	14.86	18.75	15.16
Smoking (current)	6.79	16.36	4.76	16.57	4.03	14.86	1.88	15.16
Smoking (unknown)	0.00	16.36	0.00	16.57	0.00	14.86	0.00	15.16
White blood cells [10*3/ul]	7.33 (4.21, 13.11)	0.51	7.78 (4.23, 13.22)	0.19	8.26 (4.75, 14.57)	0.21	8.64 (5.04, 15.27)	-
Neutrophil [%]	72.48 (55.56, 84.16)	0.93	73.25 (58.11, 85.00)	0.76	75.45 (59.00, 86.34)	0.42	77.31 (64.19, 87.00)	0.16
Lymphocytes [%]	16.95 (7.63, 31.81)	0.93	15.47 (7.00, 29.86)	0.76	14.00 (6.27, 29.00)	0.21	12.20 (5.67, 23.27)	0.16
Eosinophil [%]	0.80 (0.00, 2.78)	2.47	0.69 (0.00, 2.67)	1.71	0.67 (0.00, 2.29)	0.64	0.80 (0.00, 2.38)	0.16
Basophil [%]	0.30 (0.10, 0.60)	2.98	0.25 (0.00, 0.67)	1.33	0.05 (0.00, 0.60)	0.85	0.13 (0.00, 0.50)	0.16
Platelets [10*3/ul]	245.00 (147.69, 392.20)	0.51	235.74 (143.95, 375.16)	0.19	233.82 (125.74, 388.84)	0.21	267.77 (136.48, 412.06)	-
C-reactive protein [mg/l]	80.13 (13.00, 182.56)	15.23	71.94 (11.95, 173.43)	18.29	99.96 (11.19, 222.68)	12.53	115.41 (42.10, 208.91)	0.78
hs. C-reactive protein [mg/l]	83.87 (6.17, 176.70)	93.42	56.14 (4.96, 158.78)	-	99.13 (21.72, 190.01)	92.57	53.05 (3.15, 184.19)	87.19
Procalcitonin [ng/ml]	0.17 (0.05, 2.39)	51.95	0.16 (0.04, 2.38)	50.86	0.26 (0.04, 5.18)	20.59	0.33 (0.05, 4.78)	3.44
Fibrin D-dimer [mg/l]	0.89 (0.26, 4.72)	-	0.84 (0.36, 6.26)	87.81	0.79 (0.21, 9.20)	96.60	0.56 (0.36, 0.75)	99.69
Ferritin [ng/ml]	585.12 (111.20, 2187.44)	17.80	565.00 (117.86, 2193.25)	4.57	653.21 (99.01, 2788.20)	12.10	1067.22 (263.83, 3360.76)	0.62
Cardiac Troponin T [ng/ml]	0.01 (0.00, 0.16)	22.02	0.01 (0.00, 0.16)	16.19	0.02 (0.01, 0.40)	16.56	0.09 (0.01, 0.52)	5.00
Creatinine [mg/dl]	0.92 (0.60, 2.30)	0.72	0.93 (0.62, 2.78)	0.19	0.93 (0.58, 3.40)	-	1.00 (0.63, 3.59)	-
Lactate dehydrogenase [U/l]	318.00 (182.06, 603.50)	22.43	346.50 (198.26, 581.00)	8.00	349.57 (192.00, 641.37)	3.82	427.31 (265.62, 725.60)	0.62
GGT [U/l]	108.50 (28.00, 821.00)	98.35	59.00 (12.60, 338.60)	93.33	54.50 (14.57, 318.00)	-	64.04 (16.25, 298.83)	65.94
AAT [U/l]	36.50 (18.00, 88.00)	4.42	38.63 (20.00, 99.79)	1.14	49.50 (20.00, 142.00)	-	56.38 (28.00, 145.58)	0.78
Creatine kinase [U/l]	113.00 (35.50, 709.40)	51.13	125.17 (35.25, 689.00)	19.81	166.50 (39.60, 1178.00)	42.89	163.00 (42.84, 1024.13)	11.88
Bilirubin [mg/dl]	0.50 (0.30, 1.00)	4.32	0.54 (0.33, 1.09)	1.14	0.60 (0.30, 1.26)	-	0.59 (0.35, 1.20)	0.62
Albumin [g/dl]	3.30 (2.50, 4.10)	4.22	3.18 (2.44, 3.92)	1.14	2.79 (1.99, 3.74)	-	3.09 (2.20, 3.77)	0.62
IL-6 [pg/ml]	3.94 (3.08, 4.79)	99.79	29.00 (7.05, 130.00)	84.38	25.00 (8.00, 94.03)	53.72	24.33 (7.00, 171.30)	-
pH	7.40 (7.28, 7.48)	61.73	7.41 (7.27, 7.48)	48.38	7.40 (7.23, 7.47)	34.82	7.39 (7.25, 7.46)	22.50
PCO_2 [mmHg]	41.67 (31.00, 55.09)	62.45	41.24 (32.92, 55.76)	61.14	45.58 (29.00, 64.14)	71.34	43.22 (29.92, 60.08)	52.03
PaO_2 [mmHg]	83.10 (61.00, 113.28)	62.35	88.60 (62.10, 132.71)	61.33	103.01 (58.55, 147.42)	71.34	99.28 (63.60, 141.65)	52.03
HCO_3 [mmol/l]	25.00 (20.00, 30.19)	52.47	25.40 (20.02, 29.82)	30.29	24.93 (19.25, 30.00)	32.48	24.77 (19.26, 30.74)	22.03
CO_2 [mmol/l]	24.67 (21.00, 28.71)	5.35	24.20 (20.09, 27.78)	0.38	25.00 (20.77, 29.00)	0.21	24.11 (19.76, 28.05)	-

Supplementary Table 8: Descriptive statistics for selected subgroups consisting of held-out Optum test cohort patients with no more than a certain number of covariates missing in their EHRs (Less than 6 missing, Less than 9 missing). Input covariates of CovEWS are placed towards the bottom of the table and separated from covariates that are not inputs by a horizontal line. For binary covariates, the Value columns indicate the percentage of patients presenting with the condition at the end of their respective observation periods. For continuous measurements, the Value columns indicate the median and 10th and 90th percentiles in parentheses of the observed value for measurements that are collected once per patient, such as age, and the median of observed values for measurements that are collected multiple times per patient, such as heart rate. BMI = Body Mass Index, HIV = Human Immunodeficiency Virus, COPD = Chronic Obstructive Pulmonary Disease, GGT = Gamma Glutamyl Transferase, AAT = Aspartate Aminotransferase, IL6 = Interleukin 6, n/a = not available

		Optum Test Set March 21 - June 5 2020			
		Less than 6 missing covariates		Less than 9 missing covariates	
		Value	Miss.%	Value	Miss.%
	Patients [#]	1 301	-	2 872	-
	COVID-19 [%]	100.00	-	100.00	-
	Hispanic [%]	8.99	-	10.93	-
	Black [%]	27.82	-	28.20	-
	Caucasian [%]	45.66	-	46.17	-
	Asian [%]	5.30	-	4.18	-
	Hospital admission [%]	59.42	-	59.68	-
	ICU admission [%]	31.05	-	22.46	-
	Mortality [%]	23.75	-	17.51	-
Model Inputs	Female [%]	39.82	-	43.80	-
	Age [years]	66.00 (46.00, 83.00)	-	65.00 (43.00, 84.00)	-
	Weight [kg]	85.56 (61.56, 120.84)	1.38	83.91 (60.36, 120.10)	3.24
	Height [cm]	167.64 (154.94, 182.88)	3.77	167.64 (154.94, 182.88)	6.48
	BMI [kg/m^2]	29.36 (22.75, 41.62)	1.69	29.39 (22.30, 41.37)	3.93
	Intubation [%]	32.44	-	19.01	-
	Temperature [C]	36.90 (36.49, 37.53)	3.31	36.88 (36.50, 37.50)	3.83
	SpO_2 [%]	94.89 (92.36, 97.25)	0.31	94.99 (92.64, 97.44)	0.70
	Heart rate [/min]	86.51 (71.86, 102.56)	1.61	85.16 (70.65, 101.20)	2.79
	Respiratory rate [/min]	21.55 (18.07, 27.00)	1.92	20.17 (17.87, 25.93)	3.20
	Dyspnea [%]	79.55	-	75.63	-
	Sys. blood pressure [mmHg]	122.26 (107.53, 139.99)	1.31	123.29 (107.89, 141.75)	2.47
	Dias. blood pressure [mmHg]	69.38 (60.05, 80.29)	1.31	70.68 (60.44, 82.00)	2.51
	Kidney disease [%]	32.97	-	30.15	-
	Ischemic heart disease [%]	40.81	-	36.39	-
	Other heart diseases [%]	86.78	-	83.50	-
	Cerebovascular disease [%]	21.52	-	20.19	-
	Hypertension [%]	76.02	-	72.95	-
	Diabetes [%]	49.19	-	46.59	-
	Hyperlipidemia [%]	59.72	-	57.59	-
	Cancer [%]	24.67	-	24.90	-
	COPD [%]	16.30	-	16.89	-
	Asthma [%]	16.99	-	18.38	-
	Pulmonary embolism [%]	8.92	-	6.86	-
	Connective tissue disease [%]	4.46	-	4.63	-
	Inflamatory bowel disease [%]	2.00	-	1.57	-
	Osteoarthritis [%]	30.44	-	30.95	-
	Rheumatroid arthritis [%]	32.74	-	32.00	-
	HIV [%]	0.92	-	0.91	-
	Smoking (never)	62.18	6.76	61.11	8.60
	Smoking (previous)	26.83	6.76	25.17	8.60
	Smoking (current)	4.23	6.76	5.12	8.60
	Smoking (unknown)	0.00	6.76	0.00	8.60
	White blood cells [10*3/ul]	9.32 (5.42, 16.16)	-	8.23 (4.65, 14.51)	-
	Neutrophil [%]	78.62 (65.72, 87.27)	-	76.24 (61.00, 86.45)	0.03
	Lymphocytes [%]	11.26 (5.33, 21.97)	-	13.50 (5.94, 26.38)	0.03
	Eosinophil [%]	0.69 (0.00, 2.56)	-	0.62 (0.00, 2.48)	0.28
	Basophil [%]	0.20 (0.00, 0.57)	0.23	0.22 (0.00, 0.62)	0.42
	Platelets [10*3/ul]	254.29 (140.15, 397.58)	-	244.50 (142.01, 386.56)	-
	C-reactive protein [mg/l]	106.32 (38.07, 210.09)	1.31	88.57 (18.00, 200.52)	3.52
	hs. C-reactive protein [mg/l]	74.25 (8.27, 163.13)	82.17	66.00 (7.30, 159.87)	85.27
	Procalcitonin [ng/ml]	0.39 (0.06, 5.80)	23.14	0.21 (0.04, 3.16)	32.69
	Fibrin D-dimer [mg/l]	1.76 (0.49, 8.59)	77.63	1.25 (0.36, 7.36)	80.08
	Ferritin [ng/ml]	867.05 (218.16, 3020.01)	0.46	692.58 (151.73, 2594.80)	2.72
	Cardiac Troponin T [ng/ml]	0.03 (0.01, 0.42)	3.07	0.02 (0.01, 0.24)	8.46
	Creatinine [mg/dl]	1.09 (0.65, 4.07)	-	1.00 (0.63, 3.48)	-
	Lactate dehydrogenase [U/l]	400.44 (242.20, 688.24)	1.31	359.50 (217.50, 632.00)	5.26
	GGT [U/l]	67.62 (18.00, 318.11)	83.70	59.33 (15.79, 308.97)	87.50
	AAT [U/l]	49.96 (24.98, 131.57)	0.08	43.00 (21.65, 112.20)	0.17
	Creatine kinase [U/l]	155.80 (38.00, 1031.07)	14.45	131.00 (36.41, 812.83)	22.04
	Bilirubin [mg/dl]	0.59 (0.34, 1.21)	-	0.55 (0.31, 1.10)	0.03
	Albumin [g/dl]	2.90 (2.15, 3.58)	-	3.10 (2.30, 3.80)	0.07
	IL-6 [pg/ml]	30.00 (7.94, 188.95)	67.33	25.00 (7.00, 171.90)	79.53
	pH	7.40 (7.27, 7.47)	0.61	7.40 (7.28, 7.47)	32.21
	PCO_2 [mmHg]	41.65 (30.84, 55.09)	15.83	41.22 (30.00, 55.70)	47.98
	PaO_2 [mmHg]	89.91 (61.00, 131.79)	15.83	88.20 (60.37, 130.95)	48.02
	HCO_3 [mmol/l]	24.84 (19.70, 30.31)	0.38	25.00 (19.74, 30.00)	17.69
	CO_2 [mmol/l]	24.40 (20.04, 28.60)	0.31	24.29 (20.14, 28.32)	0.87

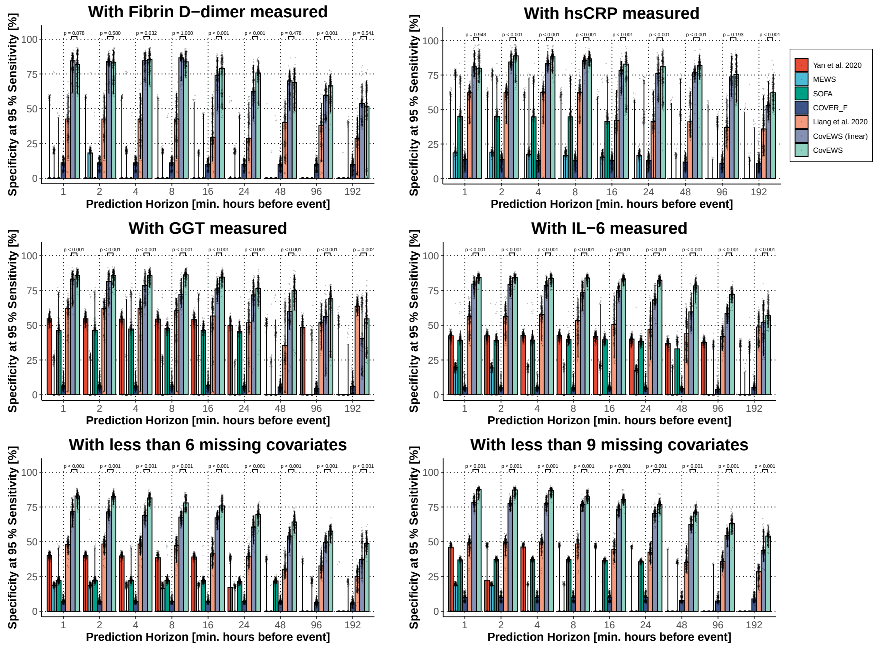

Supplementary Figure 6: Performance comparison in terms of Specificity at greater than either 90% (topmost row) or 95% (other rows) Sensitivity (y-axis) for different prediction horizons ahead of observed mortality events (in hours, x-axis) for CovEWS (light green), CovEWS (linear; light purple), Liang et al. (orange) [18], COVID-19 Estimated Risk for Fatality (COVER_F; blue) [19], Sequential Organ Failure Assessment (SOFA; green) [14], Modified Early Warning Score (MEWS; turquoise) [23], and Yan et al. (red) [17] on subcohorts of the held-out Optum test set consisting of patients for which certain covariates were available at least once in their EHR (”With [COVARIATE_NAME] measured”) and patients with no more than 6 and 9 covariates in total not available in their EHR (”With less than [NUMBER] missing covariates”). Some methods do not reach 90% and 95% sensitivity for some horizons, and may therefore not be visible in all plots. Bars indicate median and error bars indicate 95% confidence intervals (CIs) obtained via bootstrapping with 200 samples. Detailed results are available in Section “Performance Evaluation”. One-sided Mann-Whitney-Wilcoxon tests were used to derive p values shown at the top of each plot for superiority of CovEWS over CovEWS [linear].

CovEWS Receiver Operating Characteristic (Optum Test Set)

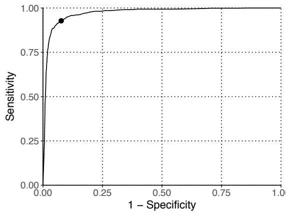

(a) 1 hour

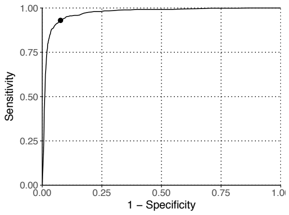

(b) 2 hours

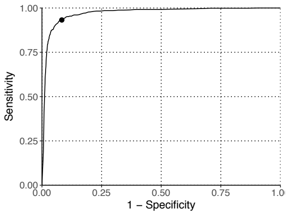

(c) 4 hours

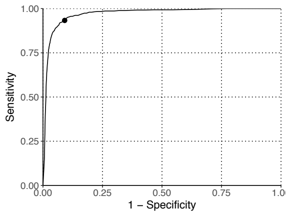

(d) 8 hours

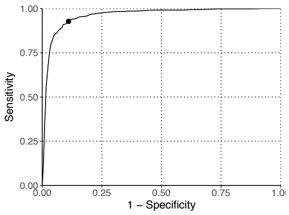

(e) 16 hours

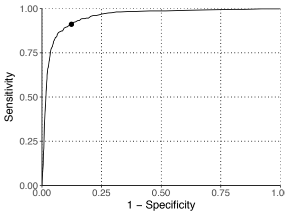

(f) 24 hours

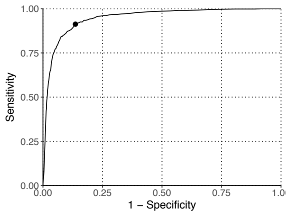

(g) 48 hours

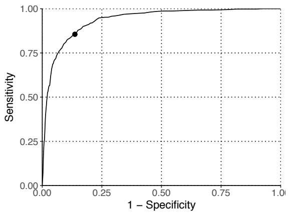

(h) 96 hours

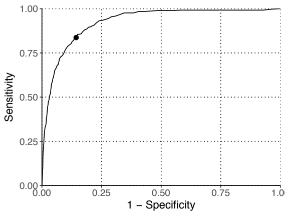

(i) 192 hours

Supplementary Figure 7: Receiver operating characteristic (ROC) curves for CovEWS for various prediction horizons between 1 and 192 hours evaluated on the held-out Optum test set. The black dot indicates the optimal decision threshold for each prediction horizon selected on the Optum validation set as the closest point on the ROC curve to the top left coordinate (closest-to-top-left heuristic).

CovEWS Receiver Operating Characteristic (TriNetX)

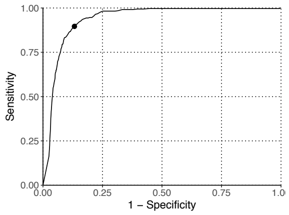

(a) 1 hour

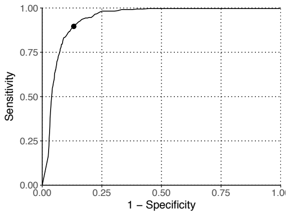

(b) 2 hours

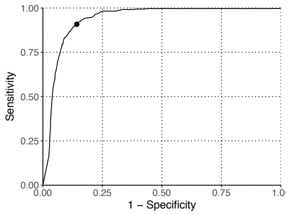

(c) 4 hours

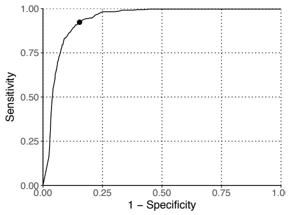

(d) 8 hours

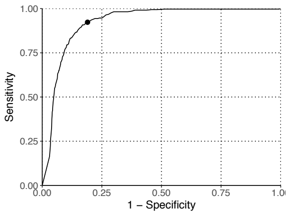

(e) 16 hours

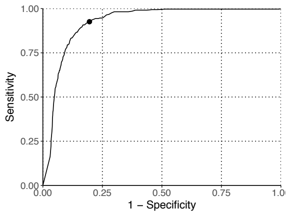

(f) 24 hours

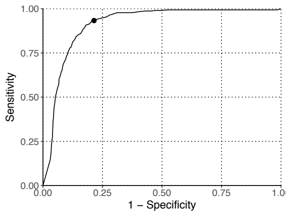

(g) 48 hours

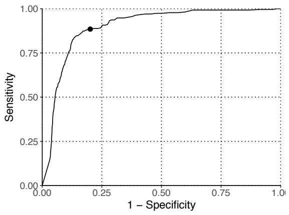

(h) 96 hours

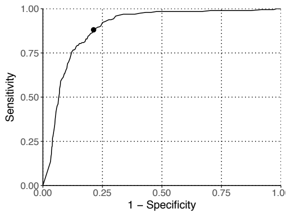

(i) 192 hours

Supplementary Figure 8: Receiver operating characteristic (ROC) curves for CovEWS for various prediction horizons between 1 and 192 hours evaluated on the external TriNetX test set. The black dot indicates the optimal decision threshold for each prediction horizon selected on the Optum validation set as the closest point on the ROC curve to the top left coordinate (closest-to-top-left heuristic).

CovEWS (linear) Receiver Operating Characteristic (Optum Test Set)

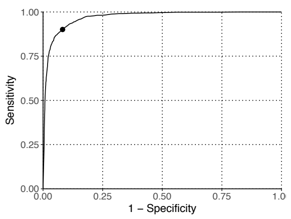

(a) 1 hour

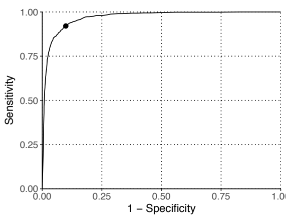

(b) 2 hours

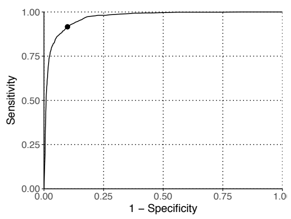

(c) 4 hours

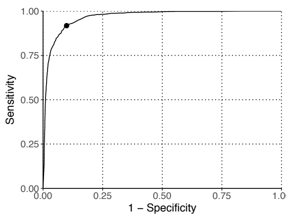

(d) 8 hours

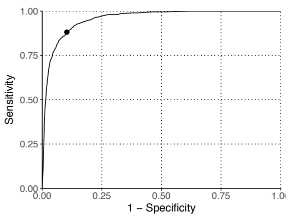

(e) 16 hours

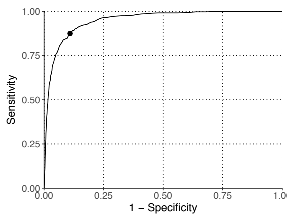

(f) 24 hours

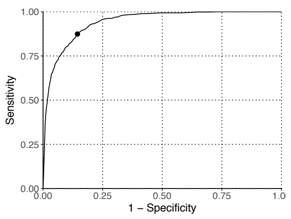

(g) 48 hours

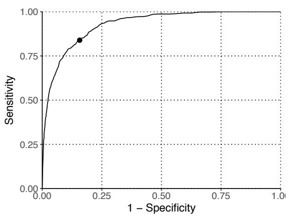

(h) 96 hours

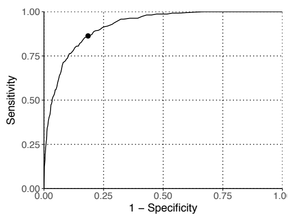

(i) 192 hours

Supplementary Figure 9: Receiver operating characteristic (ROC) curves for CovEWS (linear) for various prediction horizons between 1 and 192 hours evaluated on the held-out Optum test set. The black dot indicates the optimal decision threshold for each prediction horizon selected on the Optum validation set as the closest point on the ROC curve to the top left coordinate (closest-to-top-left heuristic).

CovEWS (linear) Receiver Operating Characteristic (TriNetX)

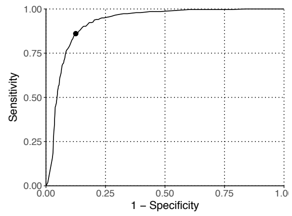

(a) 1 hour

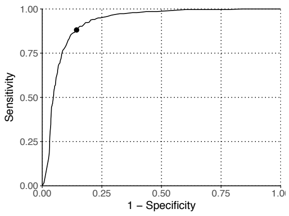

(b) 2 hours

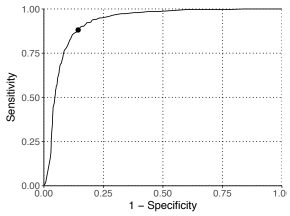

(c) 4 hours

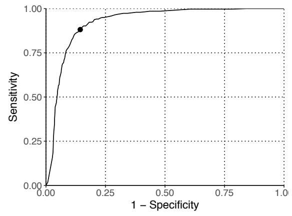

(d) 8 hours

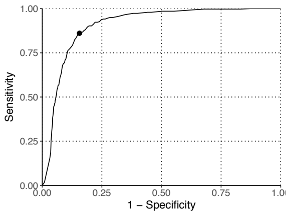

(e) 16 hours

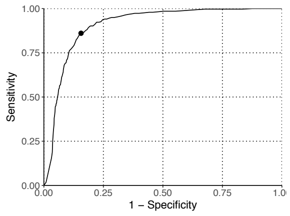

(f) 24 hours

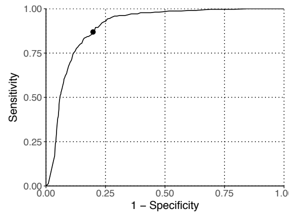

(g) 48 hours

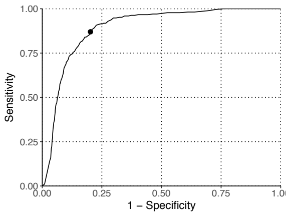

(h) 96 hours

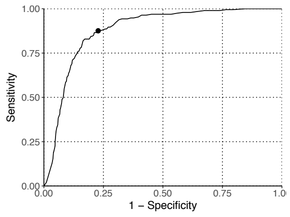

(i) 192 hours

Supplementary Figure 10: Receiver operating characteristic (ROC) curves for CovEWS (linear) for various prediction horizons between 1 and 192 hours evaluated on the external TriNetX test set. The black dot indicates the optimal decision threshold for each prediction horizon selected on the Optum validation set as the closest point on the ROC curve to the top left coordinate (closest-to-top-left heuristic).
