# Supplementary material for: Real-time prediction of COVID-19 related mortality using electronic health records
Source: Nat Commun. 2021 Feb 16;12:1058. doi: 10.1038/s41467-020-20816-7 (PMC7886884; doi:10.1038/s41467-020-20816-7)
Supplement: Supplementary file 2 — Description of Additional Supplementary Files [file 41467_2020_20816_MOESM2_ESM.docx]

**Supplementary Data 1:**

Comparison of CovEWS, CovEWS (linear) [20], COVER_F [19], SOFA [14], MEWS [23], Yan et al. [17], and Liang et al. [18] at various prediction horizons in terms of AUC, AUPR, F1, sensitivity, specificity and specificity at greater than 95% sensitivity (Spec.@95%Sens.) for predicting COVID-19 related mortality on the held-out Optum test set. Values are the median and the 95% confidence intervals (CIs, in parentheses) obtained via bootstrap resampling with 200 samples. * = significant at p < 0.05 to CovEWS (one-sided Mann-Whitney-Wilcoxon, Bonferroni corrected).

**Supplementary Data 2:**

Comparison of CovEWS, CovEWS (linear) [20], COVER_F [19], SOFA [14], MEWS [23], Yan et al. [17], and Liang et al. [18] at various prediction horizons in terms of AUC, AUPR, F1, sensitivity, specificity and specificity at greater than 95% sensitivity (Spec.@95%Sens.) for predicting COVID-19 related mortality on the external TriNetX test set. Values are the median and the 95% confidence intervals (CIs, in parentheses) obtained via bootstrap resampling with 200 samples. * = significant at p < 0.05 to CovEWS (one-sided Mann-Whitney-Wilcoxon, Bonferroni corrected).

**Supplementary Data 3:**

Comparison of CovEWS, CovEWS (linear) [20], COVER_F [19], SOFA [14], MEWS [23], Yan et al. [17], and Liang et al. [18] at various prediction horizons in terms of AUC, AUPR, F1, sensitivity, specificity and specificity at greater than 95% sensitivity (Spec.@95%Sens.) for predicting COVID-19 related mortality on the Optum future cohort. Values are the median and the 95% confidence intervals (CIs, in parentheses) obtained via bootstrap resampling with 200 samples. * = significant at p < 0.05 to CovEWS (one-sided Mann-Whitney-Wilcoxon, Bonferroni corrected).

**Supplementary Data 4:**

Comparison of CovEWS, CovEWS (linear) [20], COVER_F [19], SOFA [14], MEWS [23], Yan et al. [17], and Liang et al. [18] at various prediction horizons in terms of AUC, AUPR, F1, sensitivity, specificity and specificity at greater than 95% sensitivity (Spec.@95%Sens.) for predicting COVID-19 related mortality on the Black or African American subgroup of the Optum test set. Values are the median and the 95% confidence intervals (CIs, in parentheses) obtained via bootstrap resampling with 200 samples. * = significant at p < 0.05 to CovEWS (one-sided Mann-Whitney-Wilcoxon, Bonferroni corrected).

**Supplementary Data 5:**

Comparison of CovEWS, CovEWS (linear) [20], COVER_F [19], SOFA [14], MEWS [23], Yan et al. [17], and Liang et al. [18] at various prediction horizons in terms of AUC, AUPR, F1, sensitivity, specificity and specificity at greater than 95% sensitivity (Spec.@95%Sens.) for predicting COVID-19 related mortality on the Hispanic subgroup of the Optum test set. Values are the median and the 95% confidence intervals (CIs, in parentheses) obtained via bootstrap resampling with 200 samples. * = significant at p < 0.05 to CovEWS (one-sided Mann-Whitney-Wilcoxon, Bonferroni corrected).

**Supplementary Data 6:**

Comparison of CovEWS, CovEWS (linear) [20], COVER_F [19], SOFA [14], MEWS [23], Yan et al. [17], and Liang et al. [18] at various prediction horizons in terms of AUC, AUPR, F1, sensitivity, specificity and specificity at greater than 95% sensitivity (Spec.@95%Sens.) for predicting COVID-19 related mortality on the Asian subgroup of the Optum test set. Values are the median and the 95% confidence intervals (CIs, in parentheses) obtained via bootstrap resampling with 200 samples. * = significant at p < 0.05 to CovEWS (one-sided Mann-Whitney-Wilcoxon, Bonferroni corrected).

**Supplementary Data 7:**

Comparison of CovEWS, CovEWS (linear) [20], COVER_F [19], SOFA [14], MEWS [23], Yan et al. [17], and Liang et al. [18] at various prediction horizons in terms of AUC, AUPR, F1, sensitivity, specificity and specificity at greater than 95% sensitivity (Spec.@95%Sens.) for predicting COVID-19 related mortality on the Caucasian subgroup of the Optum test set. Values are the median and the 95% confidence intervals (CIs, in parentheses) obtained via bootstrap resampling with 200 samples. * = significant at p < 0.05 to CovEWS (one-sided Mann-Whitney-Wilcoxon, Bonferroni corrected).

**Supplementary Data 8:**

Comparison of CovEWS, CovEWS (linear) [20], COVER_F [19], SOFA [14], MEWS [23], Yan et al. [17], and Liang et al. [18] at various prediction horizons in terms of AUC, AUPR, F1, sensitivity, specificity and specificity at greater than 95% sensitivity (Spec.@95%Sens.) for predicting COVID-19 related mortality on the non-hospitalised subgroup of the Optum test set. Values are the median and the 95% confidence intervals (CIs, in parentheses) obtained via bootstrap resampling with 200 samples. * = significant at p < 0.05 to CovEWS (one-sided Mann-Whitney-Wilcoxon, Bonferroni corrected).

**Supplementary Data 9:**

Comparison of CovEWS, CovEWS (linear) [20], COVER_F [19], SOFA [14], MEWS [23], Yan et al. [17], and Liang et al. [18] at various prediction horizons in terms of AUC, AUPR, F1, sensitivity, specificity and specificity at greater than 95% sensitivity (Spec.@95%Sens.) for predicting COVID-19 related mortality on the Fibrin D-dimer subgroup of the Optum test set. Values are the median and the 95% confidence intervals (CIs, in parentheses) obtained via bootstrap resampling with 200 samples. * = significant at p < 0.05 to CovEWS (one-sided Mann-Whitney-Wilcoxon, Bonferroni corrected).

**Supplementary Data 10:**

Comparison of CovEWS, CovEWS (linear) [20], COVER_F [19], SOFA [14], MEWS [23], Yan et al. [17], and Liang et al. [18] at various prediction horizons in terms of AUC, AUPR, F1, sensitivity, specificity and specificity at greater than 95% sensitivity (Spec.@95%Sens.) for predicting COVID-19 related mortality on the hsCRP subgroup of the Optum test set. Values are the median and the 95% confidence intervals (CIs, in parentheses) obtained via bootstrap resampling with 200 samples. * = significant at p < 0.05 to CovEWS (one-sided Mann-Whitney-Wilcoxon, Bonferroni corrected).

**Supplementary Data 11:**

Comparison of CovEWS, CovEWS (linear) [20], COVER_F [19], SOFA [14], MEWS [23], Yan et al. [17], and Liang et al. [18] at various prediction horizons in terms of AUC, AUPR, F1, sensitivity, specificity and specificity at greater than 95% sensitivity (Spec.@95%Sens.) for predicting COVID-19 related mortality on the Gamma Glutamyl Transferase subgroup of the Optum test set. Values are the median and the 95% confidence intervals (CIs, in parentheses) obtained via bootstrap resampling with 200 samples. * = significant at p < 0.05 to CovEWS (one-sided Mann-Whitney-Wilcoxon, Bonferroni corrected).

**Supplementary Data 12:**

Comparison of CovEWS, CovEWS (linear) [20], COVER_F [19], SOFA [14], MEWS [23], Yan et al. [17], and Liang et al. [18] at various prediction horizons in terms of AUC, AUPR, F1, sensitivity, specificity and specificity at greater than 95% sensitivity (Spec.@95%Sens.) for predicting COVID-19 related mortality on the IL-6 subgroup of the Optum test set. Values are the median and the 95% confidence intervals (CIs, in parentheses) obtained via bootstrap resampling with 200 samples. * = significant at p < 0.05 to CovEWS (one-sided Mann-Whitney-Wilcoxon, Bonferroni corrected).

**Supplementary Data 13:**

Comparison of CovEWS, CovEWS (linear) [20], COVER_F [19], SOFA [14], MEWS [23], Yan et al. [17], and Liang et al. [18] at various prediction horizons in terms of AUC, AUPR, F1, sensitivity, specificity and specificity at greater than 95% sensitivity (Spec.@95%Sens.) for predicting COVID-19 related mortality on the less than 6 missing covariates subgroup of the Optum test set. Values are the median and the 95% confidence intervals (CIs, in parentheses) obtained via bootstrap resampling with 200 samples. * = significant at p < 0.05 to CovEWS (one-sided Mann-Whitney-Wilcoxon, Bonferroni corrected).

**Supplementary Data 14:**

Comparison of CovEWS, CovEWS (linear) [20], COVER_F [19], SOFA [14], MEWS [23], Yan et al. [17], and Liang et al. [18] at various prediction horizons in terms of AUC, AUPR, F1, sensitivity, specificity and specificity at greater than 95% sensitivity (Spec.@95%Sens.) for predicting COVID-19 related mortality on the less than 9 missing covariates subgroup of the Optum test set. Values are the median and the 95% confidence intervals (CIs, in parentheses) obtained via bootstrap resampling with 200 samples. * = significant at p < 0.05 to CovEWS (one-sided Mann-Whitney-Wilcoxon, Bonferroni corrected).
